# A Reappraisal of the Accuracy of the Tactile Method for the Detection of the Subgingival Cementoenamel Junction: An *In Vivo* Study

**DOI:** 10.1055/s-0044-1786865

**Published:** 2024-05-17

**Authors:** Jaruta Mokhagul, Attawood Lertpimonchai, Lakshman Samaranayake, Orawan Charatkulangkun

**Affiliations:** 1 Department of Periodontology, Faculty of Dentistry, Chulalongkorn University, Bangkok, Thailand; 2 Center of Excellence in Periodontal Disease and Dental Implant, Faculty of Dentistry, Chulalongkorn University, Bangkok, Thailand; 3 Faculty of Dentistry, University of Hong Kong, Hospital Road, Hong Kong

**Keywords:** accuracy, cementoenamel junction, clinical attachment level, detection

## Abstract

**Objectives** This article reappraises the accuracy and factors associated with the detection of the cementoenamel junction (CEJ) using the tactile method.

**Materials and Methods** A total of 111 tooth sites of 7 patients scheduled for flap surgery were selected for the study. The CEJ was detected in a blind manner using the conventional tactile method with a standard periodontal probe by a single, trained examiner. A custom-made stent was prepared to standardize the measurements and the distance from a fixed reference point on the stent to the CEJ was measured before (apparent CEJ) and after (real CEJ) opening a gingival flap. To evaluate the effect of local anesthesia (LA) on the measurement error, assessment with and without LA given prior to the measurement was also evaluated. The bone crest-CEJ distance at each site was also recorded in all sites.

**Statistical Analysis** The measurement error of apparent versus real distance, if any, was compared using Cohen's weighted kappa coefficient (WKC) (± 1 mm).

**Results** A weak WKC (WKC = 0.539) was found between the apparent and real CEJ distance. Higher WKCs were noted at posterior and proximal sites than the anterior and buccal/lingual sites, respectively (0.840 and 0.545 vs. 0.475 and 0.488). A higher confluence of the agreements was noted when CEJ distance was measured in anesthetized sites (WKC = 0.703). Sites without bone loss showed more coronal deviation of CEJ detection, as opposed to apical deviation seen at sites with bone loss.

**Conclusion** The conventional CEJ detection using the tactile method was relatively imprecise depending on the anatomical location of the tooth and the bone loss at the site of measurement. However, the detection accuracy improved when the sites were anesthetized. In clinical terms, our data, reported here for the first time imply that, in the absence of visual cues, posterior tooth site measurements of periodontal attachment loss were more reliable in comparison to the other sites. The bone crest level also impacted the measurement deviation to some extent, implying that, possible overestimate of clinical attachment loss may occur at sites without bone loss.

## Introduction

Periodontitis is an inflammatory disease of the supporting tissues surrounding the teeth. The disease is essentially caused by the destruction of periodontal tissues due to an excessive host immune response to plaque biofilm, resulting in the periodontal attachment loss, pocket formation, and eventually alveolar bone loss. Thus, the measurement of the clinical attachment loss (CAL) is routinely used to evaluate the degree of periodontal disease in a given site. Indeed, the most recent World Workshop on staging and grading of periodontitis uses the attachment loss as a key measure for classifying periodontitis. 1

The major anatomical landmark used for measuring the attachment loss is the cementoenamel junction (CEJ). The CEJ thus acts as a fixed reference point and is a key parameter for diagnosing periodontitis and monitoring disease improvement or remission posttreatment. In healthy teeth the CEJ, located subgingivally, is measured using tactile sense in the absence of a direct visual field. Thus, the time-honored technique for identifying the CEJ is the tactile method where a calibrated periodontal probe is inserted into the gingival sulcus/periodontal pocket to detect the irregular margin between the enamel and cementum. The periodontal probe can thus be used to measure the distance from the CEJ to the bottom of sulcus/pocket, from which the CAL can be derived. 2 Clearly, this method has inherent inadequacies which are fairly well explained in the early literature. 3 4 However, contemporary investigations on the validity of clinical assessments of the location of the CEJ using the tactile method are sparse and somewhat contradictory. 4 5

There are multiple factors confounding the accurate measurement of the CEJ including the tooth anatomy, and its location as well as the alveolar bone crest level and the fixed reference point used for these measurements. 4 Patient discomfort/pain upon inserting a periodontal probe beneath the gum may also affect the accuracy of detecting the CEJ. In addition, no reports are available on either the effect of patient discomfort/pain or the alveolar bone crest level on the accuracy of CEJ detection.

Therefore, the aims of the current study were to determine the accuracy of detecting the CEJ by a periodontal probe using the tactile method by comparing the measurement variation, if any, under a closed approach (apparent length) and an open flap approach (real length), as well as before and after local anesthesia (LA). Hence, using a custom-made acrylic splint, we evaluated the following factors affecting the accuracy of CEJ estimation: the tooth anatomy, and its location, the impact of LA, and the alveolar bone crest level.

## Materials and Methods

### Study Population

A total of seven subjects (two with gingivitis and five with periodontitis) were recruited from the Graduate Periodontics Clinic, Bangkok, Thailand. The inclusion criteria were patients who had completed the hygienic phase of periodontal disease management and scheduled for periodontal flap surgery. All selected patients had teeth with a subgingival CEJ in the targeted surgical areas and sites in these areas were carefully examined and selected for the evaluations. The exclusion criteria were sites with cervical carious or noncarious lesions, cervical restorations, subgingival calculus, and cementoenamel projection found before or after a flap opening.

The sample size was calculated with a significance level alpha of 5% and power of 80%. A sample size of 110 sites was required with a 30% likelihood of excluded sites. The subjects who met the inclusion criteria were contacted and informed about the study purpose and procedures, and those willing to participate in the study provided signed consent.

The study protocol was approved by the Ethics Committee of where the research was conducted (HREC-DCU 2021-017) and in accordance with the Helsinki Declaration of 1975, as revised in 2013.

### Examination Equipment

**Fig. 1:**
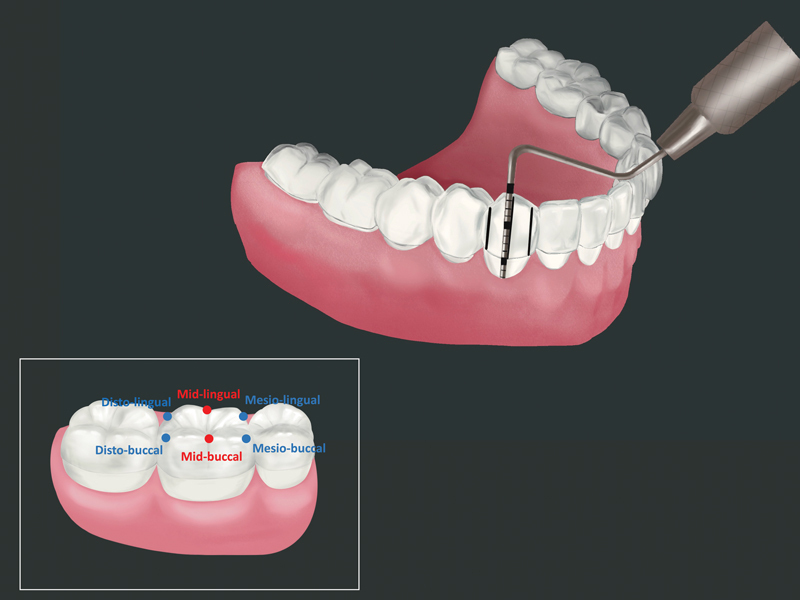
Custom-made vacuum-formed clear stent with black lines at six sites on the examined teeth, used as the fixed-references for cementoenamel junction (CEJ) measurement. The box on left indicates the examined tooth sites, i.e., mesio-buccal/mid-buccal/disto-buccal/mesio-lingual/mid-lingual/disto-lingual.

A 0.5-mm-thick custom-made vacuum-formed clear stent (Duran, Scheu Dental GmbH Am Burgberg, Iserlohn, Germany) with colored markings was fabricated to obtain a fixed reference for CEJ measurement. For this purpose, study cast of each patient was obtained and a full-arch with full-crown coverage stent was fabricated. The stents were cut and trimmed at the selected tooth sites leaving coronal-half coverage of the clinical crown, permitting the CEJ to be examined. Black marker lines were drawn on the stent at six sites (mesio-buccal/mid-buccal/disto-buccal/mesio-lingual/mid-lingual/disto-lingual) around the examined teeth. These markers were used as the fixed reference points for inserting the periodontal probe so that repeat measurements were performed using the identical positions. The lower border of the stent was used as the reference point for measuring the distance from the stent to the CEJ ( Fig. 1 ). The stent was evaluated for stability prior to the examination.

All examinations were performed using the University of North Carolina periodontal probe (UNC 15, Hu-Friedy, Chicago, Illinois, United States) with 1-mm scale markings.

### Cementoenamel Junction Detection and Examination

All the examinations were performed by a single trained clinician. Three examinations of a specific site were performed: the first and second examinations were performed using a closed approach, before and after giving LA, and the final examination was done with an open flap approach. To reduce any recall bias of the examiner, the first examination was carried out at least 1 day prior to the surgery. The second and third examinations were performed at the same visit but approximately 30 to 45 minutes apart.

To determine the CEJ location, the distance from the lower border of the stent to the CEJ at each site was measured. The CEJ location was identified by the change in the tactile sensation felt by the examiner when the probe was inserted apically from the coronal to the apical (root) direction, guided by the stent and the tooth surface. The transition point of the smooth surface of the enamel to the rough surface of the cementum was taken as the landmark for measurement of the CEJ. In the open approach, the CEJ was visually detected as it was clearly visible to the examiner due to the raised gingival flap, and the measurement was relatively straight forward.

The sites with an undetectable CEJ using the closed approach were noted and the recorded distance was measured from the stent to the bottom of sulcus/pocket. Furthermore, to investigate whether the alveolar crest level affected the accuracy of CEJ measurement, the distance from the CEJ to the bone crest was also recorded during the open approach once the flap was raised.

Prior to the experiment, the examiner (J.M.) was trained thoroughly, and the concordance of measurement performed by an experienced periodontist and the examiner was correlated. In the event, a very high concordance for the intraexaminer reliability was noted, with a weighted kappa score of 0.93.

### Statistical Analysis

Cohen's weighted kappa coefficient (WKC) (within ± 1 mm) was used to compare the agreement of the CEJ location examined between before (both with and without LA) and after flap opening and in each subgroup of the bone crest to CEJ distance evaluation.

The difference between the apparent CEJ distance and the real CEJ distance was determined and presented as a frequency distribution. Statistical analyses were performed using IBM SPSS Statistic version 28.0 for Windows (IBM Corporation, Armonk, New York, United States). A WKC of agreement level were: 0 to 0.20 as none; 0.21 to 0.39 as minimal; 0.40 to 0.59 as weak; 0.60 to 0.79 as moderate; 0.80 to 0.90 as strong; and > 0.90 as almost perfect. 6

## Results

Of the seven subjects enrolled in the study, two had gingivitis and five had periodontitis. In total, 111 tooth sites of the 7 subjects with subgingivally located CEJs were evaluated. These comprised 30 anterior teeth (27%) and 81 posterior teeth sites (73%). Among these, 71 (64%) and 40 sites (36%) were proximal and buccal/lingual sites, respectively. As per CEJ-bone crest level determination, 25 sites (23%) comprised 1-mm group, 65 sites (58%) 2- to 3-mm group, and finally, 21 sites (19%) 4- to 6-mm group ( Table 1 ).

**Table 1:** Periodontal case diagnosis of subjects and characteristic of the study sites

Case diagnosis	Total study sites, *N* (%)	Proximal site, *N* (%)	Buccal/lingual site, *N* (%)	Sites on anterior teeth, *N* (%)	Sites on posterior teeth, *N* (%)	CEJ-bone distance		
						1 mm, *N* (%)	2–3 mm, *N* (%)	4–6 mm, *N* (%)
Gingivitis ( *N* = 2)	27 (24)	17 (15)	10 (9)	−	27 (24)	15 (14)	12 (11)	−
Periodontitis stage I grade A ( *N* = 2)	32 (29)	21 (19)	11 (10)	12 (11)	20 (18)	8 (7)	22 (19)	2 (2)
Periodontitis stage I grade B ( *N* = 1)	28 (25)	18 (16)	10 (9)	18 (16)	10 (9)	2 (2)	19 (17)	7 (6)
Periodontitis stage III grade C ( *N* = 2)	24 (22)	15 (14)	9 (8)	−	24 (22)	−	12 (11)	12 (11)
Total ( *N* = 7)	111 (100)	71 (64)	40 (36)	30 (27)	81 (73)	25 (23)	65 (58)	21 (19)

Abbreviation: CEJ, cementoenamel junction.

### CEJ Detection Accuracy

**Fig. 2:**
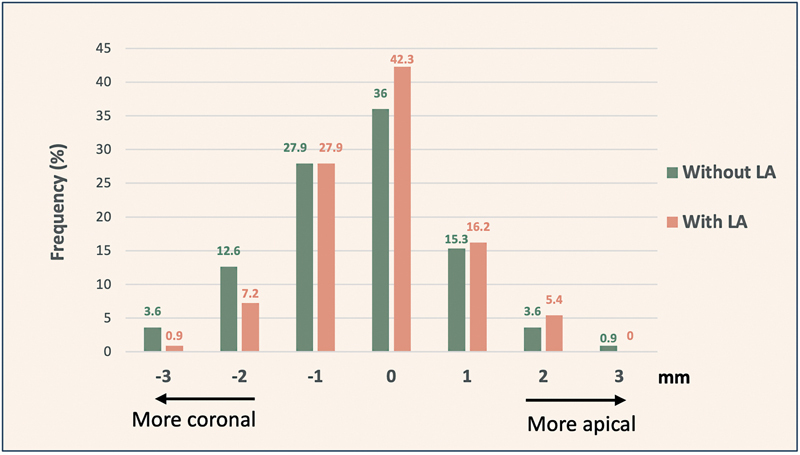
Frequency distribution of variations in cementoenamel junction (CEJ) distance measured before and after flap opening both with and without local anesthesia (LA).

The WKC comparing the CEJ location measurements before and after flap opening was 0.539 with a 79.28% agreement (weak agreement). Of these, 16.2% of sites were identified as coronal while 4.5% were apical to the real CEJ location. However, this concurrence for CEJ detection was stronger for the posterior teeth and relatively weak for the anterior teeth with WKC of 0.840 and 0.475, respectively. Additionally, the WKC of the proximal sites were relatively higher than that for the buccal/lingual sites, 0.545 and 0.488, respectively ( Table 2 and Fig. 2 ).

**Table 2:** The real and apparent evaluation of subgingival CEJ relative to (1) the anatomical location of the tooth, (2) site of the tooth, and (3) CEJ-bone crest distance

Study sites		Without local anesthesia			With local anesthesia		
		Kappa score a	Agreement	Level of agreement	Kappa score a	Agreement	Level of agreement
**Total**		**0.539**	**79.28%**	**Weak**	**0.703**	**86.49%**	**Moderate**
Tooth position	Anterior	0.475	56.7%	Weak	0.669	73%	Moderate
	Posterior	**0.840**	87.7%	**Strong**	**0.884**	91%	**Strong**
Tooth site	Proximal	0.545	81.69%	Weak	**0.796**	91.55%	**Strong**
	Buccal/lingual	0.488	75%	Weak	0.540	77.5%	Weak
CEJ-bone crest distance	1 mm	0.495	84%	Weak	0.701	92%	Moderate
	2–3 mm	0.533	76.9%	Weak	0.756	87.7%	Moderate
	4–6 mm	0.548	80.95%	Weak	0.541	80.95%	Weak

Abbreviations: CEJ, cementoenamel junction; LA, local anesthesia.

Note: The percent agreement and the weighted kappa coefficients of measurements before (with and without LA) and after flap opening is shown. No significance value is available for this statistic analysis. The boldfaced was used to emphasize the results of total unit and the strong agreement results.

a Cohen's weight kappa (within ± 1 mm).

On evaluation of whether the relative differences in the detected CEJ measurements were more apically or coronally skewed (both before and after flap opening) we noted that the measurements diverged 0 to 3 mm in both in the apical and coronal directions of the real CEJ ( Fig. 2 ). Indeed, an accurate CEJ was reported at the exact location only in 36% of the sites while a maximum 3-mm deviation from the real location was found in 5.4% of the examined sites.

### Accuracy of the CEJ Detection Under LA

The WKC for the CEJ detection between examination under LA before flap opening and after flap opening was 0.703 with an 86.49% agreement. The WKC for CEJ examination under LA was greater than without LA. Additionally, the accuracy of CEJ detection at the proximal sites improved from weak to strong under LA, with a WKC of 0.545 and 0.796, respectively ( Table 2 ).

### Accuracy of the CEJ Detection in Relation to the Distance from the Bone Crest to the CEJ

The study sites were divided into three groups according to the distance from the bone crest to the CEJ as, (1) 1 mm, (2) 2 to 3 mm, and (3) 4 to 6 mm. In each group, the concordance of agreement of measurements before and after flap opening was compared. In the event, the highest WKC was found in the 4- to 6-mm group, while the 1-mm group demonstrated the lowest WKC, 0.548 and 0.495, respectively ( Table 2 ). However, the WKC for all three groups was weak.

The WKC of the CEJ examination with LA in the 1-mm group and 2- to 3-mm group were 0.701 and 0.756, respectively, which were greater than examinations without LA (0.495 and 0.533, respectively). This implies a level of agreement improvement from weak to moderate in both groups due to examinations performed after LA. Nevertheless, such an increased WKC before and after LA was not seen in the 4- to 6-mm group.

**Fig. 3:**
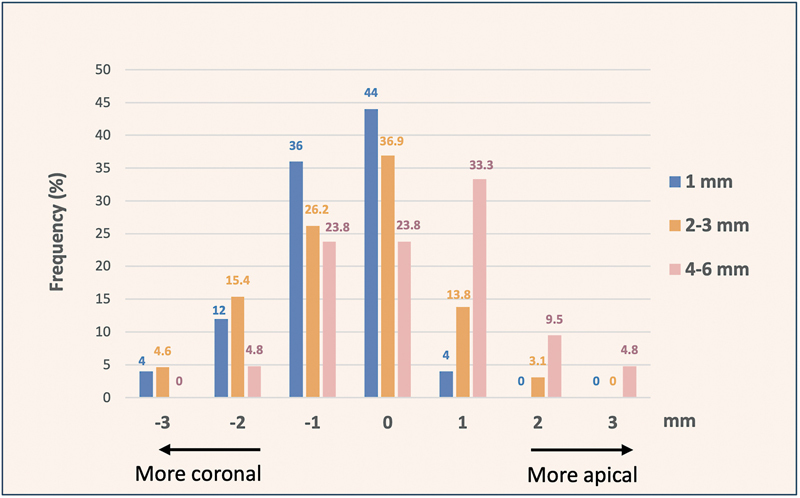
Frequency distribution of variations in cementoenamel junction (CEJ) distance measured before and after flap opening, either apical or coronal deviation, relative to CEJ-bone crest distance. The CEJ-bone crest distance was compartmentalized into three subgroups: (a) 1 mm, (b) 2–3 mm, and (c) 4–6 mm.

Finally, with regards to the detection of the deviation of the CEJ, we noted mostly a coronal deviation in the 1-mm and 2- to 3-mm group, in comparison to the 4- to 6-mm group, where the CEJ was predominantly detected apical to its location ( Fig. 3 ).

## Discussion

In this study we evaluated the accuracy of subgingival CEJ detection via the traditionally used tactile method using a periodontal probe under a number of different clinical scenarios: namely, before and after opening a periodontal flap, and with and without LA. The concordance of agreement of these measurements performed by a single trained operator was then assessed by computation of Cohen's WKC. Such stringent statistical analyses have not been performed by previous workers in the field. 4

We noted, in general, the level and concordance of agreement in detecting the anatomical delineation of the subgingival CEJ within a ± 1-mm margin of error using the traditional periodontal probe to be weak with a WKC of 0.539 (0.475–0.796). Nevertheless, it could be stated that the divergence in the accuracy of CEJ detection was accentuated more on the proximal than the buccal/lingual tooth sites, and this observation was stronger for posterior teeth, than for the anterior teeth ( Table 2 ). These findings concur with one of the earliest, seminal studies on the subject done by Hug et al in the United States over half a century ago. 4 They were the first to report (with a very low correlation coefficient of 0.37) that a periodontal probe may not be able to accurately locate the subgingival CEJ.

Using a method similar to ours, Vandana and Gupta 5 used a custom-made stent to compare the accuracy of the subgingival CEJ location before and after flap opening, using a UNC-15 periodontal probe. They too noted that the deviation in CEJ measurement was more skewed coronally than apically (43.6% vs. 31%), a result similar to ours. However, most of their remaining outcomes were discordant with our findings. Thus, they found no significant difference between the measurements of CEJ before and after flap opening. One reason for this could be the statistical analysis, the paired *t* -test, they used in comparison to the WKC evaluations employed by us. The modified WKC 6 analysis we employed was very stringent and considered deviations less than ± 1 mm as not significant. Also, a kappa score below 0.60 was considered a rather dubious level of agreement and a result that should be treated with caution.

Furthermore, at sites without bone loss, the CEJ detection typically deviated more coronally than apically, and hence inaccurate measurement of attachment loss is more likely and a diagnosis of periodontitis rather than gingivitis, might be incorrectly made.

A high degree of accuracy in detecting the CEJ is important for clinical diagnosis and management of periodontal diseases. This is illustrated by the recent declarations of the 2017 World Workshop on staging and grading of periodontitis. Accordingly, the current definition of periodontitis is a detectable interdental attachment loss without any threshold, on two or more nonadjacent teeth, or buccal or oral CAL equal to or more than 3 mm with probing depth greater than 3 mm on more than two teeth. 1 Thus, accurate CEJ detection is crucial in identifying and classifying sites with CAL, particularly in early cases. This is all the more important as the threshold for differentiation between gingivitis and periodontitis has a relatively small margin of error of 1 mm as per the new classification of Tonetti et al. 1

It appears that the topography, particularly the curvature of the lateral surfaces of the teeth may also impact accuracy of CEJ identification and lead to measurement errors. Thus, we noted a greater concordance in agreement in CEJ measurements for posterior than the anterior teeth. One possible reason for this may be the bulbous, crown contours of the posterior teeth in comparison to the anterior teeth. In contrast, the virtually continuous merger of the crown enamel margin and the root cementum in the anterior teeth is likely to make this evaluation comparably more difficult to detect by tactile sensation. Similar results were found between the proximal and buccal/lingual sites, and again it is likely that the greater curvature of the CEJ at proximal sites 7 might facilitate a more accurate CEJ detection than buccal/lingual sites.

The accuracy of CEJ detection improved, from a weak to moderate WKC value, when the tissues were locally anesthetized. It is tempting to speculate that a reason for this may be the low level of patient discomfort/pain arising during the probing process when the tissue is anesthetized. Anesthesia permits the examiner to probe without hindrance of patient complaints, thus helping to ascertain the CEJ more precisely. This is particularly true in cases with firm gingival tissues or in those with dentine hypersensitivity.

Our study seems to indicate that anesthetizing the measurement sites improve the CEJ detection accuracy. However, full mouth anesthesia for the purpose of evaluating the CEJ is not a practical proposition for routine clinical examination. Nevertheless, our data points to a confounder that impedes the accurate measurement of CEJ in diagnostic dentistry.

With regards to the bone crest level to CEJ measurement, in general, we noted a weak WKC for all such evaluations. However, the measurement error decreased in the 1-mm and 2- to 3-mm bone crest-CEJ group when the subjects were anesthetized. Further, in the 4- to 6-mm bone crest-CEJ group, the agreement level remained weak, irrespective of anesthesia.

Several issues appertaining to the methodology of our study is noteworthy. Of the three major studies available in the literature (including ours) on this subject, two employed a UNC-15 periodontal probe with 1-mm scale marking, 5 while a copy of a Hu-Friedy Qulix color-coded probe was used as a conventional probe (with a flat disc at the working tip) in the third study by Hug et al. 4 Although modifying the probe tip with flat disc might help gain better tactile sensation in detecting the CEJ margin, this modification, according to their results, did not confer an additional advantage or improved the quality of CEJ measurement. Therefore, we chose a conventional periodontal probe, the universally used instrument for periodontal examination for our study.

Additionally, the fixed reference point, which is essential to ensure an identical departure point for repeated measurements, varied between the three reported studies. Whereas a 2- to 3-mm-thick acrylic stent 5 and an orthodontic arch wire bonded to enamel 4 were used by the previous workers, we employed a very thin, 0.5 mm, vacuum-formed, color-marked stent which we believe overcame the limitations faced by the pervious researchers. Clearly, a very thin thickness stent facilitates the intimate adaption of the periodontal probe to the tooth surface and reduces the measurement error.

Furthermore, we used well-defined inclusion and exclusion criteria for site selection to reduce any bias. Hence, only sites with a distinct subgingival CEJs were selected to minimize confounding detection errors due to anatomical aberrations, calculus deposits, or restorations. Other strengths of our study were the tailor-made, thin, transparent acrylic stent and the appropriate statistical analytical tool of WKC.

On the other hand, there are a few limitations to our study. First, is the highly discerning inclusion criterion of site selection with subgingival CEJ, by virtue of which our results may not be extrapolated to every tooth site. Second, due to the restrictions imposed by the stent, the proximal sites were evaluated using a line angle approach, not the mid-proximal approach which is the routine for the clinical measurement of proximal sites. Third, as all the participants had completed the hygienic phase of periodontal treatment, this would not accurately reflect the “real world” scenario with untreated periodontitis, subgingival calculus, and an inflamed periodontium.

## Conclusion

In clinical terms, our data, reported here for the first time, imply that imprecise CEJ measurements may be relatively common except perhaps in posterior tooth sites. Also, sites without bone loss may lead to an impression of excessive CAL. Clinicians should bear in mind these inherent inaccuracies and the limitations when measuring CEJ and attachment loss when charting and classifying periodontal diseases and their progress.
